# Case Report: Latent autoimmune diabetes in two young female patients successfully treated with oral semaglutide and basal insulin

**DOI:** 10.3389/fendo.2026.1833794

**Published:** 2026-06-30

**Authors:** Maria Elena Lunati, Giuseppe Galesi, Davide Bernasconi, Camilla Tinari, Paolo Fiorina

**Affiliations:** 1 Division of Endocrinology, ASST Fatebenefratelli-Sacco, Milan, Italy; 2 Department of Biomedical and Clinical Sciences, University of Milan, Milan, Italy; 3 International Center for T1D, Pediatric Clinical Research Center Romeo ed Enrica Invernizzi, DIBIC, Università di Milano, Milan, Italy; 4 Nephrology Division, Boston Children’s Hospital, Harvard Medical School, Boston, MA, United States

**Keywords:** adjunctive therapy, autoimmune diabetes, GLP-1RA, LADY, semaglutide

## Abstract

Latent autoimmune diabetes in youth (LADY) is a poorly characterized form of autoimmune diabetes with onset between 8 and 29 years of age and with slow progression toward insulin dependence, similarly to latent autoimmune diabetes of the adult (LADA), which leads to diagnostic difficulties and possibly to therapeutic implications. In this case series, we discuss two cases of juvenile- and young adult-onset autoimmune diabetes with very low insulin need at least after diagnosis and the efficacy of treatment with semaglutide. In case 1, a 20-year-old woman with obesity (31.8 kg/m^2^) with a non-ketoacidotic onset of diabetes was initially put on metformin and basal insulin. Previous ultrasound of the abdomen showed a hyperechogenic liver pattern. Tests showed high-titer multiple diabetes-related autoantibodies positivity, but still detectable C-peptide levels; thus, metformin was stopped and semaglutide was started. After 12 months, the patient still continues oral semaglutide and basal insulin, with detectable C-peptide, and has experienced significant weight loss (−20 kg) and resolution of liver steatosis. In case 2, a 13-year-old girl was diagnosed with autoimmune diabetes, with tests showing high-titer multiple diabetes-related autoantibodies positivity. She was initially put on multiple daily insulin injections (MDI). After almost 2 years, presenting to our care with still detectable C-peptide, we decided to stop meal-time insulin and to start oral semaglutide. After 15 months of follow-up on the latter treatment, the patient presents optimal glycemic control and increased C-peptide levels. This is the first case series that shows the possibility of treating LADY with oral semaglutide and basal insulin with optimal glycometabolic outcomes. Moreover, semaglutide treatment also had beneficial effects on weight control and non-confirmed effects on metabolic dysfunction-associated steatotic liver disease (MASLD).

## Introduction

In the 2019 WHO classification of diabetes, slowly evolving immune-mediated diabetes mellitus was introduced (1), as a hybrid form of diabetes with features of both type 1 diabetes (T1D) and type 2 diabetes (T2D) and within are enclosed latent autoimmune diabetes of the adult (LADA) and latent autoimmune diabetes in youth (LADY) (2). LADA accounts for 2%–12% of all patients with diabetes; it is defined as an autoimmune form of diabetes with adult onset (>30 years at diagnosis) and no insulin requirement for at least 6 months after diagnosis. It is characterized by an intermediate phenotype with autoimmunity, although less enhanced than in T1D, and insulin resistance, as in T2D (3). In contrast, LADY is poorly characterized and is currently described in patients with an age ranging from 8 to 29 years. On the spectrum between T1D and T2D, LADY is closer to T1D than LADA; in fact, LADY shows a greater autoimmunity than LADA in terms of autoantibody titer, number of autoantibodies, and epitopes recognized by autoantibodies (2). In a study including 299 children and adolescents with LADY, 52.6% of individuals were positive for two or more diabetes-related autoantibodies (4), versus a much lower prevalence, ranging from 9% to 25.7%, reported in different studies in patients with LADA (5–7). Moreover, LADY and T1D present an enhanced intramolecular epitope spreading phenomenon in contrast to LADA (2). In the context of autoimmune diabetes with partially preserved beta-cell function and slow progression to insulin deficiency, emerging data suggest the possibility of using glucagon-like peptide-1 receptor agonists (GLP-1RAs) (3). Moreover, several trials on the use of GLP-1RAs in the setting of overt T1D showed benefits in terms of glycometabolic control and weight reduction (8, 9). In this case series, we discuss two young female patients who presented to our care with a slowly progressive form of autoimmune diabetes with partially preserved beta-cell function, who successfully obtained glycemic control with no need for short-acting insulin thanks to the administration of oral semaglutide.

## Case 1

**Figure 1:**
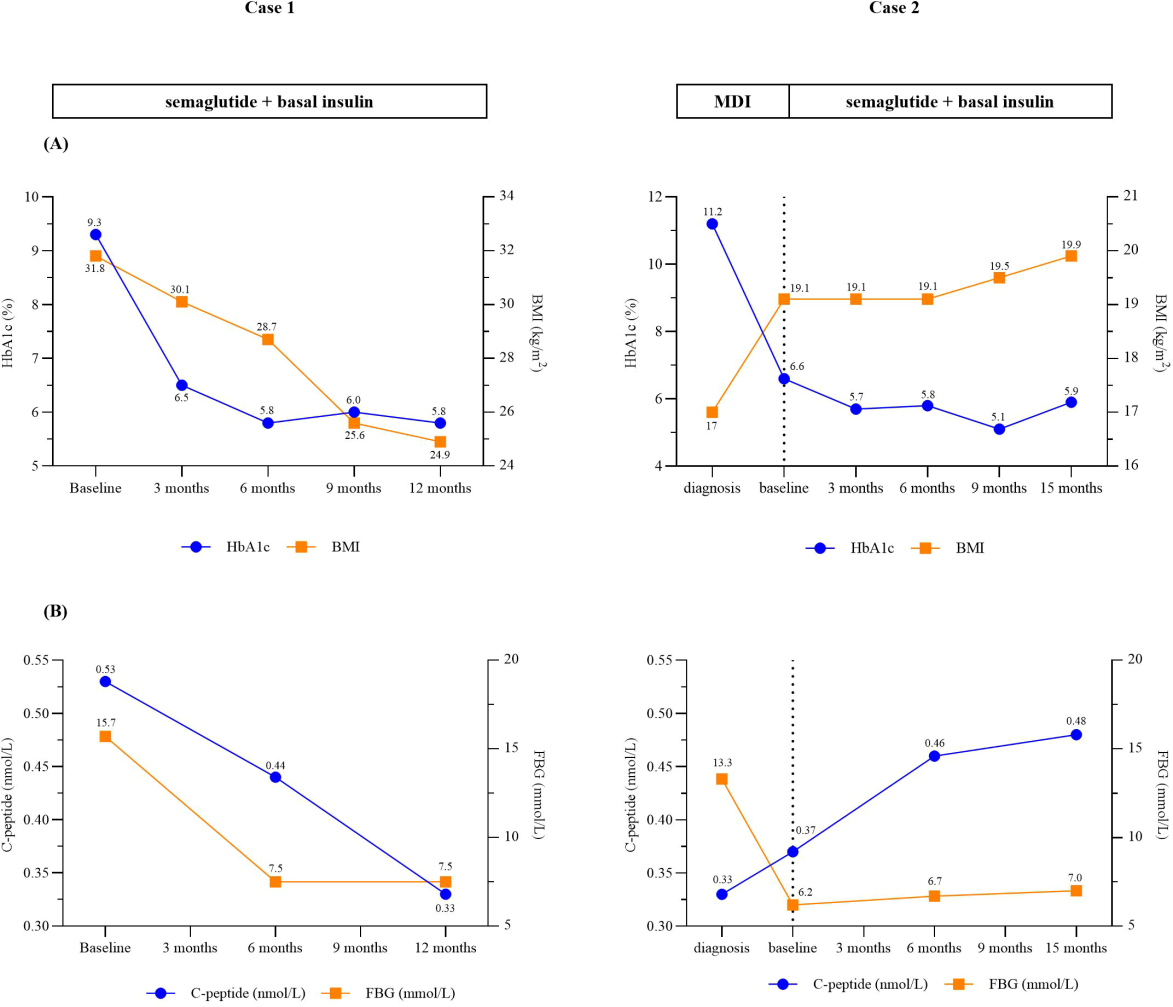
Glycometabolic parameters, C-peptide values, and fasting blood glucose before and after oral semaglutide administration. BMI, body mass index; HbA1c, glycated hemoglobin; FBG, fasting blood glucose.

**Figure 2:**
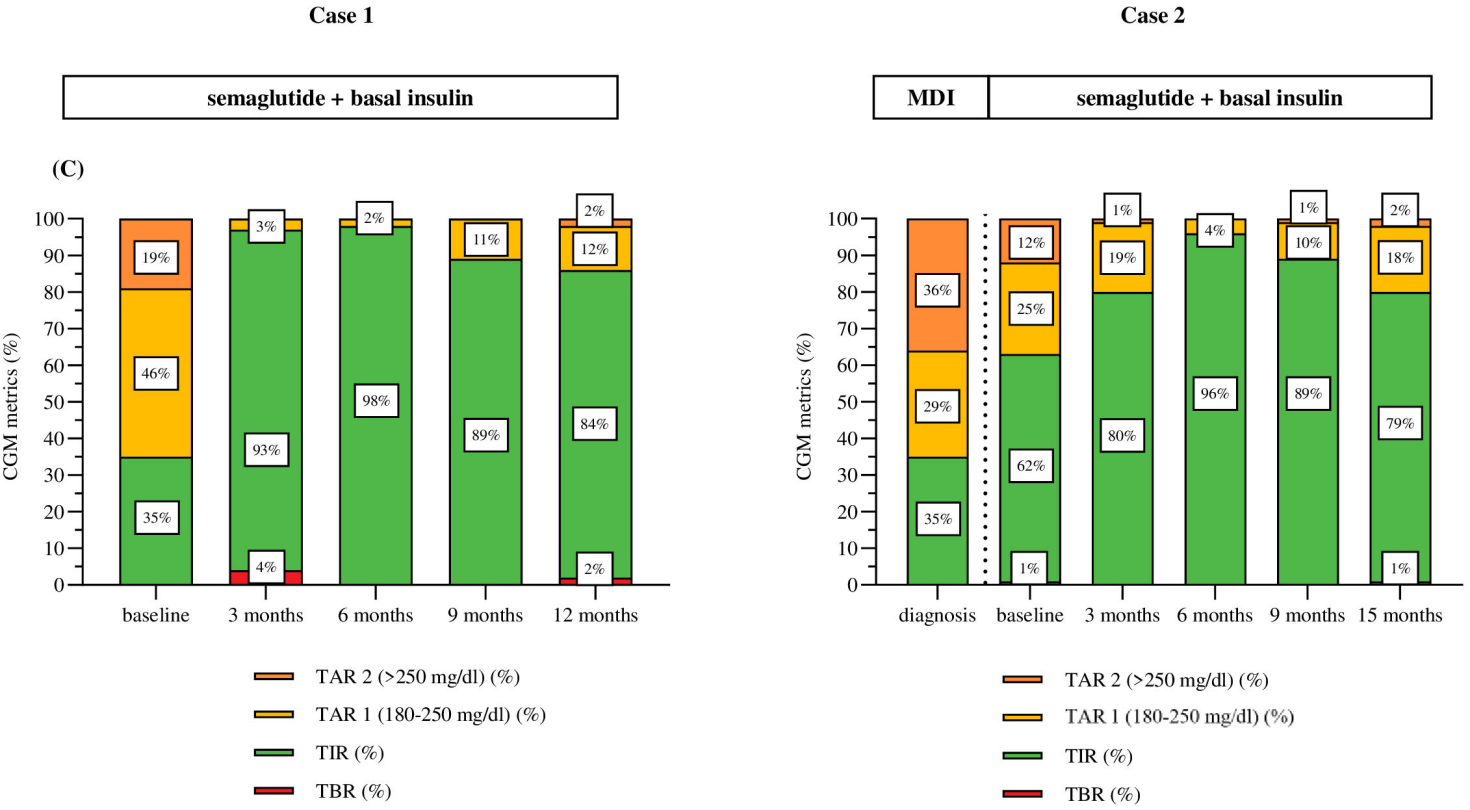
Glucose metrics before and after oral semaglutide administration. TAR 1, time above range 1 (180–250 mg/dL); TAR 2, time above range 2 (>250 mg/dL); TIR, time in range; TBR, time below range.

A 20-year-old woman [height: 170 cm; weight: 92 kg; body mass index (BMI): 31.8 kg/m^2^] presented at emergency care with hyperglycemia [peripheral blood glucose (PBG): 26.6 mmol/L], polydipsia, and polyuria. No signs of diabetic ketoacidosis (DKA) were detected [venous blood gas analysis (VBG): pH 7.36; bicarbonates: 29.4 mEq/L; potassium: 3.8 mEq/L; lactates: 1.5 mmol/L; blood glucose: 16.4 mmol/L]. After the management of acute hyperglycemia, the patient was discharged on metformin (1 g/day) and insulin degludec (8 IU/day) prescription, paired with flash glucose monitoring (FGM). Shortly after, she presented to our care. Familial history was positive for T2D while no autoimmune diseases were found in relatives. Medical history reported impaired fasting glucose [fasting blood glucose (FBG): 6.4 mmol/L], with onset 9 months before presentation, and hyperechoic liver pattern on abdominal ultrasonography observed 3 months before. The patient also reported having started following a dietetic program for weight management 2 months before the presentation to our care. Laboratory tests showed elevated glycated hemoglobin (HbA1c: 9.3%) and positivity and high titer for anti-ZnT8, anti-GAD, and anti-IA2 antibodies (**Table 1**). Fasting C-peptide measurement was found detectable (0.53 nmol/L), in the presence of fasting hyperglycemia (FBG: 15.7 mmol/L). Considering partially preserved beta-cell function, in order to achieve metabolic and weight control, oral semaglutide 3 mg/day was added on top of the already prescribed therapy. One month after the first evaluation, metformin was suspended. Subsequently, oral semaglutide was progressively titrated up to the maximally tolerated dose (14 mg/day) and basal insulin was adjusted according to fasting glycemic levels. No side effects were reported during semaglutide titration. Of note is the attempt to suspend basal insulin at 6 months after diagnosis, although the patient’s fear of glycemia elevation led to the resumption of therapy. After 1 year of follow-up (present time), the patient remains in optimal glycemic control (HbA1c 5.8%) with oral semaglutide (14 mg) and low dosage of basal insulin (10 UI, 0.14 IU/kg/day) and in bolus-free therapy (**Figures 1**, **2**). Regarding weight management, after 1 year, the patient shifted from class I obesity to normal weight (BMI at baseline vs. after 12 months: 31.8 vs. 24.9 kg/m^2^) (**Figure 1**). Moreover, the hyperechogenic liver pattern, previously reported, was not confirmed; unfortunately, other methods for further evaluation were not available at follow-up.

**Table 1:** Results of investigations at initial presentation to emergency room.

Parameters	Case 1	Case 2
Age (yrs)/Gender (F/M)	20.5/F	13.4/F
BMI (kg/m2)	31.8	17
HbA1c (mmol/mol - %)	78–9.3	99–11.2
FBG (mmol/L)	15.7	13.3
C-peptide (nmol/L)	0.53	0.33
Serum creatinine (mg/dL)	0.49	0.52
LDL-cholesterol (mg/dL)	121	144
Triglycerides (mg/dL)	120	134
HDL-cholesterol (mg/dL)	40	49
Total cholesterol (mg/dL)	185	221
AST (U/L)	18	9
ALT (U/L)	18	11
ACR (mg/g)	25	7.1
TSH (mUI/L)	1.87	1.67
Anti-thyroperoxidase Abs	–	+
Anti-thyroglobulin Abs	–	–
IgA anti-transglutaminase	–	–
Diabetes-related Abs (positivity/titer)		
GADA (IU/mL)	+/>120	+/1149
ZnT8A (U/mL)	+/>500	+/211
IA-2A (U/mL)	+/191	+/91

Yrs, years; BMI, body mass index; HbA1c, glycated hemoglobin; FBG, fasting blood glucose; LDL, low-density lipoprotein; HDL, high-density lipoprotein; AST, aspartate aminotransferase; ALT, alanine aminotransferase; TSH, thyroid-stimulating hormone; ACR, albumin–creatinine ratio; Abs, antibodies; GADA, glutamic acid decarboxylase autoantibodies; ZnT8A, zinc transporter 8 autoantibodies; IA-2A, insulinoma-associated antigen-2 autoantibodies.

## Case 2

A 13-year-old child (height: 157 cm; weight: 42 kg; BMI: 17 kg/m^2^) presented at emergency care after incidental finding of asymptomatic hyperglycemia (PBG: 18.1 mmol/L). No signs of DKA were detected (VBG: pH 7.37; bicarbonates: 22.6 mEq/L; ketonemia: 0.3 mmol/L; potassium: 4.07 mEq/L; sodium: 136.9 mEq/L; lactates: 1.31 mmol/L; blood glucose: 19.5 mmol/L), and nothing relevant was reported on physical examination. Subsequently, the patient was hospitalized and started multiple daily insulin injection (MDI) regimen paired with FGM. No family history of diabetes or autoimmune diseases was found, while medical history was silent. Laboratory tests showed elevated HbA1c (11.2%) and positivity and high titer for anti-ZnT8, anti-GAD, and anti-IA2 antibodies; in addition, anti-TPO antibodies resulted positive, with normal thyroid function (**Table 1**). Fasting C-peptide was found to be detectable (0.33 nmol/L) with a concomitant FBG level of 13.3 mmol/L. The patient was discharged with MDI treatment (basal insulin: 12 IU/day, 0.28 IU/kg/day; prandial insulin: 16 IU/day). After 21 months, at the age of 15 years old, she came to our attention. Laboratory tests and glucose metrics showed sub-optimal glycemic control and still detectable beta-cell function (**Figures 1**, **2**). Evidence of slow progression of beta-cell destruction and patient’s will to suspend short-acting insulin led to the substitution of prandial insulin with oral semaglutide (3 mg/day). To improve her adherence to therapy, daily basal insulin glargine was substituted by weekly basal insulin icodec. Considering low BMI values, semaglutide dose was not further titrated while basal insulin was adjusted according to fasting glycemic levels. After 15 months of follow-up (present time), the patient remains in optimal glycemic control (**Figures 1**, **2**) with oral semaglutide, insulin icodec (100 IU/week, 0.28 IU/kg/day), and a very low need for short-acting insulin (2 UI/meal in case of carbohydrate-rich meals, usually twice a week). Also, we found a slight increase in fasting C-peptide values compared to values at diagnosis (fasting C-peptide at diagnosis vs. after 36 months: 0.33 nmol/L vs. 0.48 nmol/L) (**Figure 1**).

## Discussion

These cases of diabetes are characterized by post-puberal non-ketoacidotic onset, by partially preserved beta-cell function, and by reduced insulin need, despite high-titer multiple diabetes-related autoantibodies positivity, which is usually associated with a rapid progression from stage 1 and 2 to stage 3 T1D (10). Considering these characteristics, the diagnosis of LADY was proposed. Although the International Society for Pediatric and Adolescent Diabetes (ISPAD) criteria for the diagnosis of the honeymoon phase (HbA1c lower than 7% and insulin dose less than 0.5 IU/kg/day) are satisfied (11), we ruled out this possibility due to the excessive duration of this state. In fact, in a study conducted on patients with a new diagnosis of T1D, mean honeymoon phase was shorter than 3 months (10); conversely, in our patients, it was prolonged for 12 and 36 months, respectively. Similarly to LADA, LADY is currently diagnosed in the presence of autoimmune diabetes, with no insulin need for at least 6 months after diagnosis, but with age of onset ranging from 8 to 29 years (2). In both our cases, age and autoimmunity profile were consistent with LADY features previously described, although the diagnostic criterion of absence of insulin need for at least 6 months after diagnosis remains unsatisfied. However, the prolonged reduced insulin need and the non-ketoacidotic onset of diabetes suggest the possibility of classifying these cases as LADY. Additionally, an important obstacle in satisfying diagnostic criteria is the difficulty of obtaining total insulin therapy suspension due to the clinician and the patient’s fear of DKA, especially in the setting of a young patient with high-titer multiple autoantibodies positivity. In **Table 2**, we provide a summary of the main characteristics and diagnostic criteria of the different types of diabetes that may be considered in the differential diagnosis when evaluating juvenile-onset diabetes. This case series has as its main outcome the efficacy of semaglutide and basal insulin combination in maintaining optimal glycemic control without the need for short-acting insulin in two young patients with LADY. GLP-1 exerts direct glycometabolic benefits through different effects: (1) stimulation of glucose-dependent release of insulin; (2) promotion of pancreatic beta cells’ survival; (3) inhibition of alpha-cell glucagon production and hepatic glucose production; and (4) enhancement of the uptake and utilization of glucose in peripheral tissues. Moreover, GLP-1 improves glucose control indirectly by reducing food intake, through delayed gastric emptying and reduction of appetite mediated by stimulation of satiety on hypothalamic neurons (12). Previous studies highlighted the possible use of GLP-1RAs in an autoimmune setting: in patients with LADA, GLP-1RAs showed comparable efficacy to patients with T2D (13), while ADJUNCT ONE and TWO trials demonstrated the efficacy of liraglutide in reducing HbA1c, insulin total daily dose (TDD), and weight compared to placebo (8) in the setting of T1D. The ADJUST-T1D trial demonstrated the efficacy of add-on treatment with semaglutide in improving glycemic control, glucose metrics, and body weight in obese patients with T1D with AID after 6 months of follow-up (14). Given the off-label use of semaglutide in LADY, both patients were fully informed and provided consent. In both cases, TDD remained at a very low dosage during follow-up, and semaglutide treatment allowed the avoidance of bolus doses in case 1 and the substantial withdrawal of bolus in case 2. The suspension of short-acting insulin represents a significant improvement in quality of life for the patient, thanks to the reduction in injection burden and risk of hypoglycemic episodes. An improvement in fasting plasma C-peptide levels was observed in the case 2 patient even after the improvement in FBG, possibly suggesting an effect beyond the resolution of glucotoxicity. The effect of GLP-1RAs on C-peptide levels has been shown to differ between patients with T1D and those with T2D. In patients with T1D, add-on treatment with GLP-1RAs showed contrasting results; in fact, trials with exenatide and dulaglutide have reported a modest increase in plasma C-peptide levels, whereas other studies have not shown that the addition of GLP-1RA prevents progressive C-peptide decline in these patients. By contrast, in patients with T2D, GLP-1RAs have been shown to improve plasma C-peptide levels, primarily by enhancing glucose-dependent insulin secretion, reducing glucotoxicity, and insulin resistance (15). On these bases, it is possible that this finding is due to the intermediate clinical and metabolic features observed in patients with LADY. Moreover, neither patient experienced DKAs or severe hypoglycemia during the follow-up. While oral semaglutide was titrated up to the maximally tolerated dose (14 mg) in case 1, it was maintained at the lowest dose of 3 mg in case 2, even though it is not recommended (16), in order to avoid weight loss. In fact, in the ADJUST trial *post-hoc* analysis (9), the use of low-dose subcutaneous semaglutide (0.25 mg/week) permitted a mean initial reduction of TDD of 17.7%. Another point of interest is the positive effect of this combination therapy on non-glycemic outcomes. Case 1 experienced a clinically significant weight loss of 20 kg at 1-year follow-up that led to the transition from class I obesity (31.8 kg/m^2^) to normal weight (24.9 kg/m^2^), in line with recent evidence of efficacy of semaglutide for weight management in T1D, as shown by the ADJUST-T1D trial (14). The efficacy and the safety of semaglutide on weight control were also demonstrated in pediatric patients with obesity in the STEP TEENS trial, while semaglutide currently lacks evidence of efficacy and safety in pediatric patients with diabetes, although other GLP-1RAs, such as exenatide, liraglutide, and dulaglutide, showedpediatric a statistically significant reduction in HbA1c in pediatric patients with T2D. (17) Another point of interest is the loss of hyperechogenicity of the liver seen in the case 1 patient. Although it is not possible to correlate this finding with architectural changes of liver structure, the beneficial effect of semaglutide on liver has already been demonstrated in obese patients with both T2D and T1D (18, 19). To our knowledge, this case series is one of the first to describe the efficacy of semaglutide add-on treatment in patients with LADY. Other case reports have described subjects with LADY treated with sulfonylureas (20), dapagliflozin (21), and MDI (22), with worse outcomes in terms of glycemic control. Consistent with the current literature, add-on therapy with GLP-1RAs seems to be highly effective in autoimmune diabetes, especially when beta-cell function is still detectable, without substantial safety concerns. Moreover, GLP-1RAs may improve metabolic syndrome features and promote weight loss in overweight/obese patients with T1D.

**Table 2:** Comparison of T2D, LADA, LADY, T1D, and MODY.

	T2D	LADA	LADY	T1D	MODY
Age at onset	Usually in adulthood	>30 years	8–29 years	Childhood/Young adulthood	Usually <25 years
BMI	Overweight/Obese	Normal/Overweight	Normal, rarely overweight	Normal, rarely overweight	Normal, rarely overweight
Clinical presentation	Features of metabolic syndrome	Features of both T1D and T2D	Features of both T1D and T2D	Prone to ketoacidosis; Possible presence of other auto-immune diseases	Dependent on the type; Possible syndromic presentation
Progression to insulin dependence	Late	At least 6 months after diagnosis	At least 6 months after diagnosis	Acute	Possible
Autoantibody profile	Negative	Positive, usually low titer and single autoantibody	Positive, usually high titer and multiple autoantibodies	Positive, usually high titer and multiple autoantibodies	Negative
Diagnosis	Based on clinical presentation and presence of metabolic syndrome features	Based on age, autoantibodies, and time to insulin dependence	Based on age, autoantibodies, and time to insulin dependence	Based on clinical presentation and autoantibodies	Based on molecular testing

T2D, type 2 diabetes; LADA, latent autoimmune diabetes of the adult; LADY, latent autoimmune diabetes in youth; T1D, type 1 diabetes; MODY, maturity onset diabetes of the young.

## Data Availability

The raw data supporting the conclusions of this article will be made available by the authors, without undue reservation.
